# Sleep in patients with disorders of consciousness characterized by means of machine learning

**DOI:** 10.1371/journal.pone.0190458

**Published:** 2018-01-02

**Authors:** Tomasz Wielek, Julia Lechinger, Malgorzata Wislowska, Christine Blume, Peter Ott, Stefan Wegenkittl, Renata del Giudice, Dominik P. J. Heib, Helmut A. Mayer, Steven Laureys, Gerald Pichler, Manuel Schabus

**Affiliations:** 1 Laboratory for Sleep, Cognition and Consciousness, & Centre for Cognitive Neuroscience (CCNS), University of Salzburg, Salzburg, Austria; 2 ITS Informationstechnik & System-Management, Salzburg University of Applied Sciences, Salzburg, Austria; 3 Department of Computer Sciences, University of Salzburg, Salzburg, Austria; 4 Coma Science Group, Cyclotron Research Centre and Neurology Department, University and University Hospital of Liège, Liège, Belgium; 5 Apallic Care Unit, Neurological Division, Albert Schweitzer Hospital Graz, Graz, Austria; Associazione OASI Maria SS, ITALY

## Abstract

Sleep has been proposed to indicate preserved residual brain functioning in patients suffering from disorders of consciousness (DOC) after awakening from coma. However, a reliable characterization of sleep patterns in this clinical population continues to be challenging given severely altered brain oscillations, frequent and extended artifacts in clinical recordings and the absence of established staging criteria. In the present study, we try to address these issues and investigate the usefulness of a multivariate machine learning technique based on permutation entropy, a complexity measure. Specifically, we used long-term polysomnography (PSG), along with video recordings in day and night periods in a sample of 23 DOC; 12 patients were diagnosed as Unresponsive Wakefulness Syndrome (UWS) and 11 were diagnosed as Minimally Conscious State (MCS). Eight hour PSG recordings of healthy sleepers (N = 26) were additionally used for training and setting parameters of supervised and unsupervised model, respectively. In DOC, the supervised classification (wake, N1, N2, N3 or REM) was validated using simultaneous videos which identified periods with prolonged eye opening or eye closure.The supervised classification revealed that out of the 23 subjects, 11 patients (5 MCS and 6 UWS) yielded highly accurate classification with an average F1-score of 0.87 representing high overlap between the classifier predicting sleep (i.e. one of the 4 sleep stages) and closed eyes. Furthermore, the unsupervised approach revealed a more complex pattern of sleep-wake stages during the night period in the MCS group, as evidenced by the presence of several distinct clusters. In contrast, in UWS patients no such clustering was found. Altogether, we present a novel data-driven method, based on machine learning that can be used to gain new and unambiguous insights into sleep organization and residual brain functioning of patients with DOC.

## Introduction

Advances in intensive care have increased the chances of surviving after severe brain injuries and lead to an ever growing number of patients surviving and awakening from coma [1]. The extent of functional impairment and thus the diagnosis is established by examining the two main components of consciousness: arousal and awareness [2, 3]. Periods of eye opening and closing are interpreted as sleep-wake cycling and indicate a state termed “Unresponsive Wakefulness Syndrome” (UWS; [4]), while additionally, signs of being “aware”, such as command following but also simple visual fixation change the diagnosis to a state termed “Minimally Conscious State” (MCS; [5]).

Beyond this, it is also acknowledged that mere behavioral indices are insufficient for reliably determining a patient’s state and thus methods relying on brain activity, such as electroencephalography (EEG) are more and more exploited In particular, an association between electrophysiological sleep patterns and clinical outcomes was already suggested earlier [6, 7] and further supported by a recent EEG-fMRI study, where patients with the ability of command following showed more organized EEG during sleep [8]. Recently, Blume and colleagues [9] have moreover shown that the integrity of patients’ circadian rhythms was related to arousal levels, which the authors suggested may be due to a better delineation of periods of sleep and wakefulness.

However a more fine-grained sleep evaluation in patients with DOC remains matter of debate with some groups considering manual sleep staging feasible [8, 10, 11], whereas others [12, 13] believe that sleep staging by established criteria, such as the American Academy of Sleep Medicine (AASM; [14]), or Rechtschaffen and Kales (R&K; [15]) is impossible and the process would require a new consensus of what to regard as N1, N2, N3 or REM.

The central issue when applying AASM criteria is finding relatively few, yet precisely defined polysomnographic (PSG) patterns, such as rapid eye movement, sleep spindles, and K-complexes. However, in DOC patients it is inherently difficult as the severe brain injuries change the topography, power, frequency and morphology of the PSG signal. The most prominent of these problems include the general slowing of EEG seen in many DOC patients (which would indicate deep sleep in healthy individuals according to AASM criteria) and the topographically often contradicting “sleep” patterns observed simultaneously on both hemispheres such as background activity of different frequencies. The situation is additionally complicated by external artifacts such as electrical noise of medical equipment, or bad EEG signal quality due to vegetative dysregulations including extensive sweating, uncontrolled eye movements, or spasms giving rise to large artefacts.

To account for the unique patterns in DOC, researchers tend to adapt the scoring criteria, for instance by varying parameters for sleep spindle detection. However no general agreement on the details leads to substantial discrepancy in the literature; for instance sleep spindles are reported to be present in 56% [16], 33% [11] or 0% [10] of UWS subjects. Correspondingly, Giubilei and colleagues [17] even report sleep patterns comparable to healthy individuals in 9 out 10 UWS patients. Furthermore, as recently shown in our previous work [12] the number of detected sleep patterns (sleep spindles and slow waves) did not statistically differ between day and night in any of the patient groups (i.e. UWS or MCS), suggesting limited sensitivity of the traditional measures in detecting circadian variation in these patients.

To increase both reliability and validity of the analysis of EEG data, quantitative signal processing or feature extraction (e.g. time-frequency analysis), is typically exploited [18]. A fully automatic analysis of sleep EEG in DOC was proposed by Malinowska and colleagues [19], where the extracted time-frequency features significantly correlated with the (behavioral) diagnosis allowing for discrimination between UWS and MCS with an accuracy of 87%. Also Noirhomme and colleagues [20] proposed an automated analysis of the background EEG in comatose patients. Despite its efficiency, such fully data-driven features are often criticized for being difficult to interpret as to their functional meaning [11] and for lacking a link with the well-established sleep criteria. As a consequence these approaches are rarely utilized by the neuroscientific community.

Despite the promising prospect that a reliable characterization of sleep in DOC patients may advance our understanding of the pathological condition and improve the diagnosis and/or prognosis, a standardized procedure for sleep assessment is out of sight. With the purpose of designing an analysis pipeline that is more robust against the known peculiarities and challenges with DOC patients’ data and at the same time linked to the standard AASM manual, we here intended to focus on quantitative EEG signal analysis combined with machine learning techniques.

Supervised machine learning, rather than relying on predefined rules, generates rules (out of provided examples) with the inherent goal to generalize beyond those examples. Thus, the standard scoring rules (AASM) are applied indirectly by first deriving a multivariate model based on polysomnographic data from healthy individuals that has previously been scored. Next, this model is evaluated in a hard test case, namely long-term EEG of DOC patients. We applied two methods: (i) a cluster analysis for a group-wise analysis (epochs are sampled from each subject, next averaged) with the aim of testing for presence of sleep-like clusters and (ii) a supervised classification for single-subject analysis (epochs are sequentially classified for each subject). As an input for the classifier we use permutation entropy as a complexity, as its robustness against environmental noise renders it more suitable for DOC analyses as compared to features based on the frequency spectrum.

## Methods

### Ethics

The experimental protocol was approved by the Ethics Committees of the Medical University of Graz and of the University of Salzburg. The study was conducted in accordance with the ethical principles of the World Medical Association. Informed written consent was obtained from all the healthy participants as well as from relatives or legal representatives of all the patients.

### Participants

We selected 23 subjects (11 MCS and 12 UWS) from the initial sample of 40 DOC patients, depending on the availability of 24hr video recordings (without missing fragments due to position change or insufficient picture quality) which accompanied the 24hr PSGs. The full data set was published here [12]. Diagnoses of patients were established using the Coma Recovery Scale-Revised [3]. The analyzed patient sample was recorded in Austria and Belgium. A detailed description, including demographic data, is provided in S1 Table.

Full night PSG (≈8h) of 26 healthy subjects (mean age = 35, SD = 10.3, 13 males) was used in order to both train and validate a classifier prior to testing it on DOC data. All healthy sleep recordings had been scored semi-automatically using Somnolyzer 24*7 by The Siesta Group [21] according to the criteria rules of the Sleep Medicine (AASM, 2007).

### Data acquisition and pre-processing

The DOC sample comprised two subgroups with the following PSG set-ups: 18 EEG placed according to the 10–20 system [22] and six physiological channels (Austrian subgroup) or 12 EEG and four physiological channels (Belgian subgroup). The healthy subjects were recorded with 22 EEG channels and four bipolar physiological channels. All data sets were recorded with a sampling rate set to 500 Hz. The fourteen channels common to all subjects (F3, Fz, F4, C3, Cz, C4, P3, Pz, P4, T3, T4, Oz, EMG, EOG) were consequently used for the combined analysis. In healthy sample we ran the supervised classification twice (14 channels vs. 26 channels) which allowed us to evaluate the influence of available number of channels on the classification performance.

Pre-processing of EEG data was performed using the Brain Vision Analyzer software (Brain Products GmbH, Gilching, Germany, version 2.0). In order to reduce the computational load, downsampling was carried out to 250 Hz. The EEG data was filtered between 1 and 30 Hz with a zero phase IIR Butterworth filter of order 4, re-referenced to a common reference. Ocular corrections were conducted using the regression-based technique [23] on the basis of bipolar vertical and horizontal EOG channels.

The 24h longitudinal recording of each DOC patient was divided into light periods, which corresponds to circadian day (named “day-time” in the following), and circadian night (termed “night-time”). The average length of day-time and night-time was respectively 10.1 hours (SD = 3.2) and 5.9 hours (SD = 0.23). Twenty three video recordings were visually screened and rated based on 5min epochs into periods of “eyes closed” (C) and “eyes-open” (O). Periods where the state of the eyes repeatedly switched between opening and closure, were scored as open-closed (O/C). A similar operationalization of sleepiness was used in Åkerstedt and colleagues [24].

Periods during which the evaluation of the state of the eyes was impossible (i.e. due to no infrared light information, wrong positioning of patient or insufficient infrared picture quality) were categorized as unscorable. In total, (across all subjects) 15% of day-time and 62% of night-time was considered as unscorable. All non-visible epochs (mainly during nights for subjects without infrared camera) were excluded from the quantitative evaluation of the classification performance.

### Permutation entropy

Permutation entropy (PE) is a measure of the irregularity of a signal and as shown in previous studies is less affected by both environmental noise [25] and eye blinks [26] than classical spectral features such as spectral power rendering it suitable for EEG analysis in DOC. It has been used to separate consciousness from unconsciousness during anesthesia [27], to distinguish UWS and MCS patients [28, 29], and recently also to show day-night changes in DOC patients [12].

PE analysis first transposes a time series into a (temporally ordered) sequence of ordinal symbols, which is followed by the calculation of the relative frequency of each of the symbol [30]. The calculation of PE involves a selection of two parameters, (i) the embedding dimension *(n)* and (ii) tau *(τ)*. The first one defines the length of symbols—how many consecutive amplitudes are compared. The lag parameter, in turn, specifies temporal separation between points in the symbol. Both are expressed in number of data points.

Based on previous studies [27, 30] the embedding dimension was kept constant (*n* = 3). For tau values we tested two different tau values as it previously has been shown that PE sensitivity to oscillatory frequency domains varies with the tau parameter [28]. In agreement with the AASM sleep staging rules, PE analysis was performed on consecutive 30s epochs. On these epochs, the PSG signal was transformed into a sequence of n-dimensional feature vectors (also referred to as epochs), where n is defined by the number of PSG channels.

### Clustering

Agglomerative hierarchical clustering is a bottom-up method producing a tree of clusters (called a dendrogram) whose hierarchy depends on the degree of similarity between observations (here n-dimensional feature vectors, called epochs). The agglomerative clustering starts with assigning each feature vector (epoch) to its own separate cluster. Using the Euclidean distance to measure similarity between clusters, the algorithm merges the two most similar clusters and updates distances to the newly formed cluster (average linkage method). This process iterates until there is only single cluster encompassing all feature vectors (epochs) [31]. By cutting off the dendrogram at different levels of dissimilarity, different numbers of clusters emerge [32].

Here we set the cut-off parameter using healthy data by selecting a cutoff value that correspond to 5 classes as we expect 5 sleep stages to be identified in healthy individuals. In order to test for the presence of a clustering pattern in DOC patients that is similar to healthy individuals we applied the same algorithm with an identical cut-off parameter to both UWS and MCS data.

**Fig 1:**
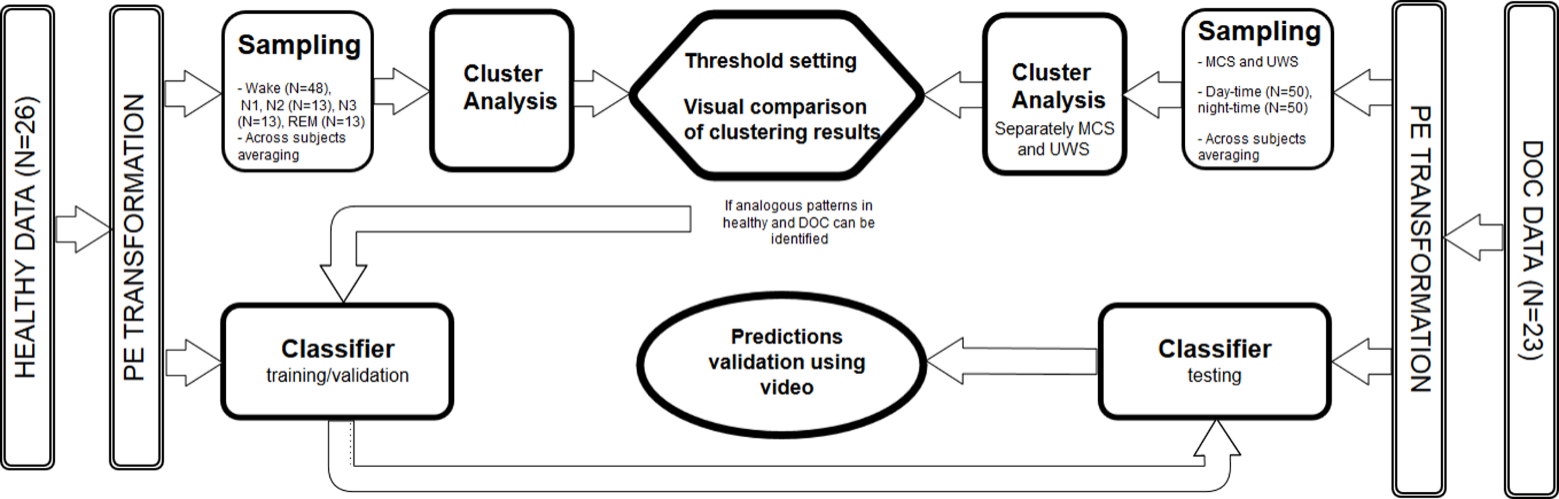
Outline of the processing pipeline. Both healthy (n = 26) and DOC (n = 23) data sets were processed by estimating signal complexity (left/right panel), next cluster analysis was applied on a group level; healthy, UWS, MCS (upper panel). Finally single subject classifier is trained using healthy previously scored data and tested on DOC, where video recordings are used as a validation proxy (lower panel). Note that first cluster analysis is used as an exploratory step. Next, after confirming that sleep patterns can be identified based on the features (PE), we train a predictive model (classifier).

The cluster-analysis was performed as a sampled, group-level analysis on epochs (30s each) averaged across subjects separately for each group (i.e. healthy, MCS, UWS) and sleep stage/circadian time. To keep the sample size consistent across groups (UWS, MCS, healthy) we randomly sampled 100 epochs (50 day-time epochs and 50 night-time epochs) from each DOC subject and 100 epochs (48 wake epochs and 13 epochs from each of the sleep stages; N1, N2, N3, REM) from each healthy subject and next averaged across subjects (cf. Fig 1).

### Classification

#### Healthy subjects analysis–training and validation of the classifier

The supervised classification was performed epoch by epoch on a single-subject level. Healthy subject data, together with standard sleep staging (five classes: wake, N1, N2, N3, REM) were used to train and validate a classifier. Specifically, a “leave one subject out cross-validation” was used such that at each iteration epochs from a single subject are reserved as a validation data set.

We compared two types of classifiers: Feedforward Neural Networks and Random Forest. The hyperparameters of the classifiers were either set as fixed (e.g. the number of trees) or by using a grid search (e.g. the number of features, the number of hidden layers) with an additional 10-fold cross validation on the training set.

The for healthy sleep typically unequal proportion of sleep stages (i.e., N2 usually occupies 50% of the total sleep) causes the classification task to be unbalanced wherefore weighted averaging of the F1-scores was used to estimate the overall (i.e. across sleep stages) classification performance. Chance level performance in healthy analysis was estimated by fitting a ‘dummy’ classifier that generates predictions based on the training set’s class distribution.

For supervised classification the F1-score was used as a performance measure [33]. The F1-score depends on both precision (ratio of true positives to all epochs predicted as positive) and recall (ratio of true positives to all positive epochs) and ranges from 0 to 1, where higher values indicate better classification performance.

#### DOC analysis–testing of the classifier

The classifier trained and optimized on healthy data was ultimately tested on the DOC dataset. We performed an epoch-by-epoch classification of PSG recordings for each patient. The output (i.e. one of 5 classes: wake, N1, N2, N3 or R) was validated by using behavioral indices of patients’ wakefulness, that is by assessing the state of the eyes based on the simultaneous video recording. The classifier’s predictions were visualized in a hypnogram, where each epoch has also been labeled with the information on the state of the eyes (i.e. open or closed). Additionally, the F1-score was used to quantify the agreement between classifiers’ predictions and behavioral markers. Note however that since the eyes only provide a binary index of patient’s wakefulness, a full validation of the classifiers’ predictions is not possible. Thus, N1, N2, N3 and REM were jointly considered as a single “sleep class” and validated against eyes closed (C).

Machine learning analyses were performed in R 3.4.0 [34] using the ‘*dendextend’* function for cluster analysis [35] and the *‘caret’* function for supervised classification [36].

## Results

### Clustering

The aim of the hierarchical cluster analysis was to check whether similar sleep-related patterns exist across groups (i.e. healthy, MCS and UWS patients). To this end, a hierarchical cluster analysis was applied separately to healthy participants’ data, MCS and UWS patients to arrive at an unsupervised grouping based on the average dissimilarities (Euclidean distance) between epochs.

**Fig 2:**
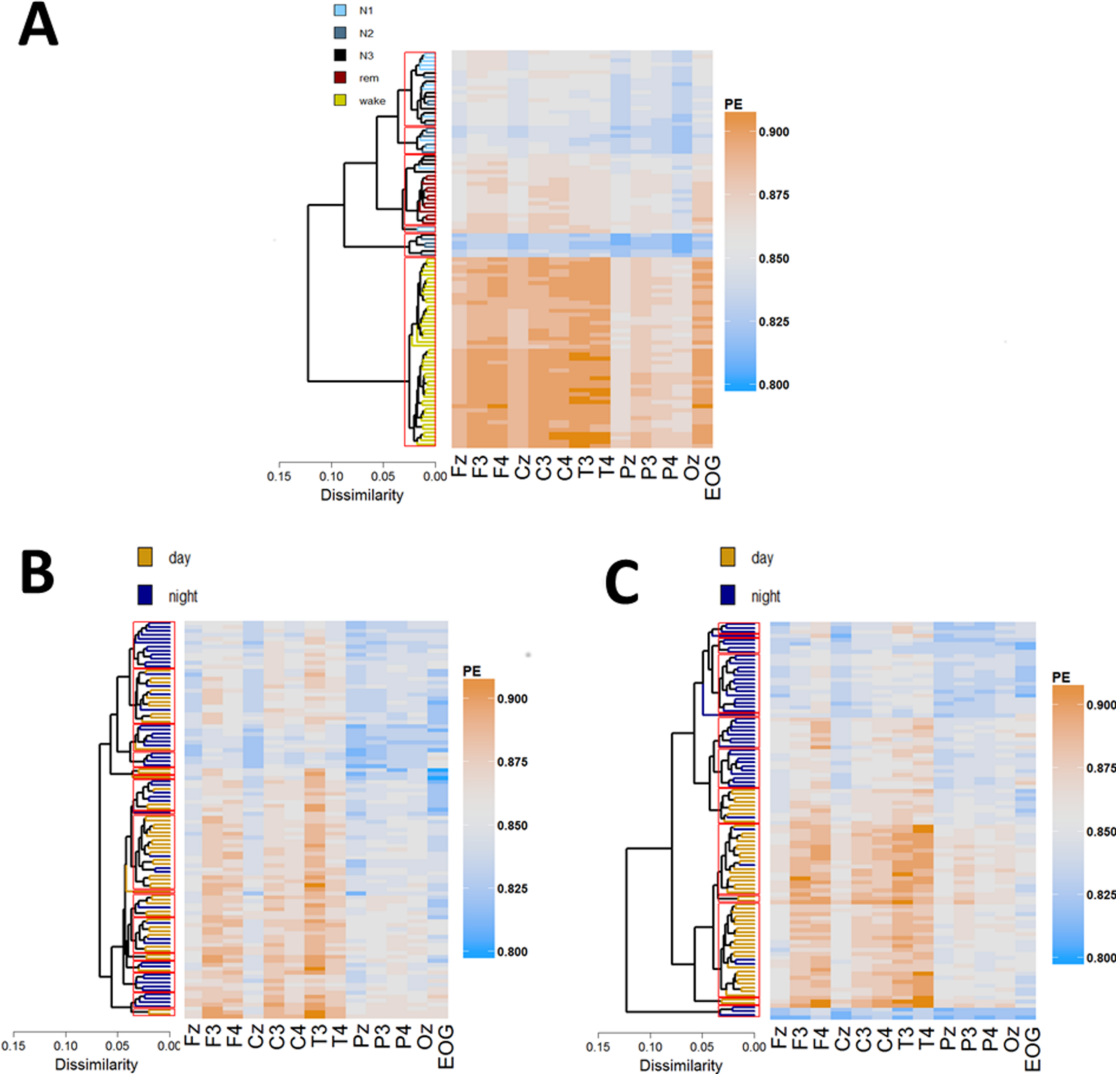
**Cluster analysis of day and night epochs for healthy (A), UWS (B) and MCS (C).** Average linkage, hierarchical clustering was applied to the rows (epochs) determining the ordering. Hot (orange) colors depict high PE values, whereas cold (blue) colors relate to low PE values across the 14 electrodes plotted on the x-axis. Cut-off parameter that corresponds to 5 sleep stages in healthy (0.035) is depicted as red rectangles. EMG was omitted from heat maps in order to enhance the visibility of differences between EEG channels. Note the clear grouping in healthy and MCS but not in UWS, which is evidenced by high inter-cluster dissimilarity (horizontal branches’ length in dendrograms).

A clustering pattern with five classes was identified in healthy individuals by thresholding at cut-off value of 0.035. We found high inter-cluster dissimilarities (Fig 2A) suggesting a clear grouping in the data. Wake sleep stage data was captured by a single cluster characterized by high entropy (mean PE = 0.884, SD = 0.003), whereas sleep clusters showed substantial overlap with standard sleep stages. Specifically, there was a single cluster of low signal complexity (uniformly distributed across channels) corresponding with N2/N3 sleep stages (mean PE = 0.830, SD = 0.003). Another cluster of relatively high complexity (especially over both EOG and fronto-central channels) was evident which corresponded predominantly with REM (mean PE = 0.862, SD = 0.003) as well as two heterogeneous clusters comprising stages N1/N2 (mean PE = 0.841, SD = 0.002) and N1/N2/N3 (mean PE = 0.850, SD = 0.003).

Similarly to healthy individuals, in MCS we found high inter-clusters dissimilarities indicating grouping in the data (cf. Fig 2, right panel). Clusters of high signal complexity (mean PE = 0.869, SD = 0.002) correspond to the day-time period, whereas clusters of relatively lower signal complexity (median PE of 0.843, SD = 0.002) coincided with the night-time period suggesting systematic day-night variation of brain activity in this group. For more details on day-night difference in these MCS as well UWS patients please also refer to Wislowska and colleagues [12]. Importantly, MCS night-time akin to healthy sleep is captured by multiple clusters. For instance we found a small, but distinct cluster characterized by not only low signal entropy (mean PE = 0.828, SD = 0.004) but also uniform distribution of PE across channels (Fig 2C) which seems to be analogous to the healthy N2-dominated cluster. We also identified clusters of comparatively high average entropy (mean PE = 0.855, SD = 0.002) (especially over frontal and temporal channels), that resembles healthy REM.

In UWS by contrast, there is only a small difference in average signal complexity between day (mean PE = 0.857, SD = 0.008) and the night-time period (mean PE = 0.852, SD = 0.01) suggesting that systematic day-night variations in brain activity are highly impaired. Moreover, cluster analyses revealed an absence of grouping as the inter-cluster dissimilarities are much smaller in UWS compared to healthy participants and MCS patients, for instance the average Euclidean dissimilarity between two most distinct clusters is 0.07 for UWS, whereas for both healthy and MCS the Euclidean dissimilarity is 0.13 and 0.12, respectively (Fig 2B, dendrograms).

### Classification in healthy

**Fig 3:**
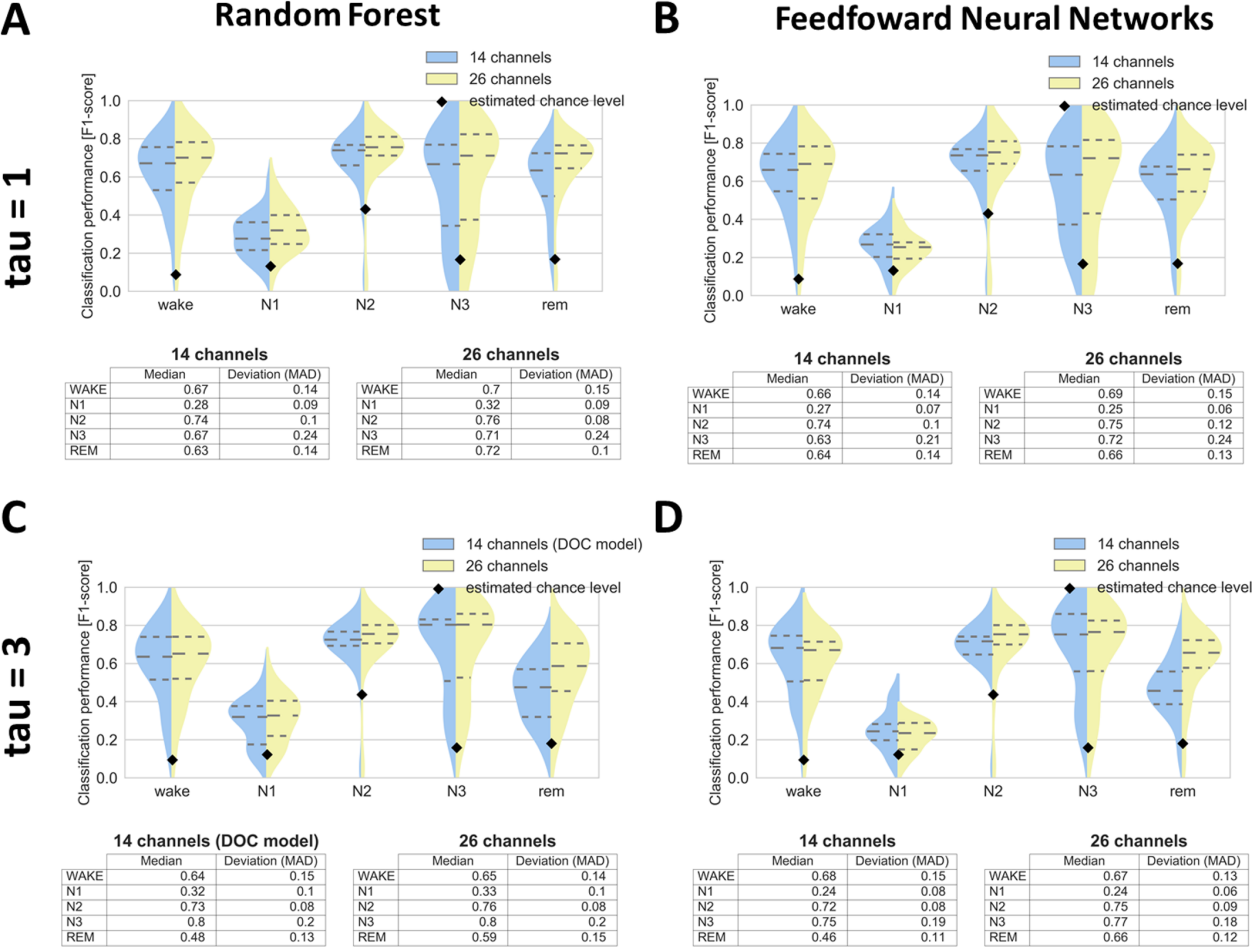
Classification of sleep stages for 26 healthy subjects (longitudinal night recordings) by using different tau parameter (rows) and different classifiers (columns). Classifiers utilizing 14 and 26 channels are colored blue and yellow respectively. Dashed horizontal lines represent quartiles whereas black diamonds chance level. Note the overall higher performance for random forest (A,C) compared to feedforward neural networks (B,D), also more acurate classification of stage N3 based on tau = 3 (C,D) compared to tau = 1 (A,B). MAD = median absolute deviation.

In order to optimize our classification setup (and thus improve generalization to DOC) we tested not only different classifiers (random forest vs. feedforward neural networks) but also two different PE parameter values (tau1 vs. tau3). Also, to evaluate the impact of reduced number of channels, we compared 26 channels setup with 14 channels setup. Random forest has proved to be slightly better than neural networks (Fig 3, compare columns), whereas the larger value of tau parameter (Fig 3, compare rows) has led to increased and comparable classification performance of sleep stage N3 and of stages N1, N2, REM respectively. Lower number of channels did not impair the classification except for REM sleep, possibly due to reduced number of physio channels in the 14 channels setup (Fig 3C, REM). In general we found that classification of stage N1, although being most difficult to classify as it is highly heterogeneous remained significantly above chance level. As the most important channels (assessed by features importance in random forest) we identified horizontal EOG, Cz, C4 and EMG, with EOG being especially useful for classifying stage N2, and EMG for classifying REM sleep.

For DOC analysis we proceeded with random forest classifier (due to its higher overall performance in healthy analysis) combined with tau = 3 (due to its higher sensitivity to N3 stage in healthy analysis) and based on 14 channels setup (Fig 3C, in blue).

### Classification in DOC

**Fig 4:**
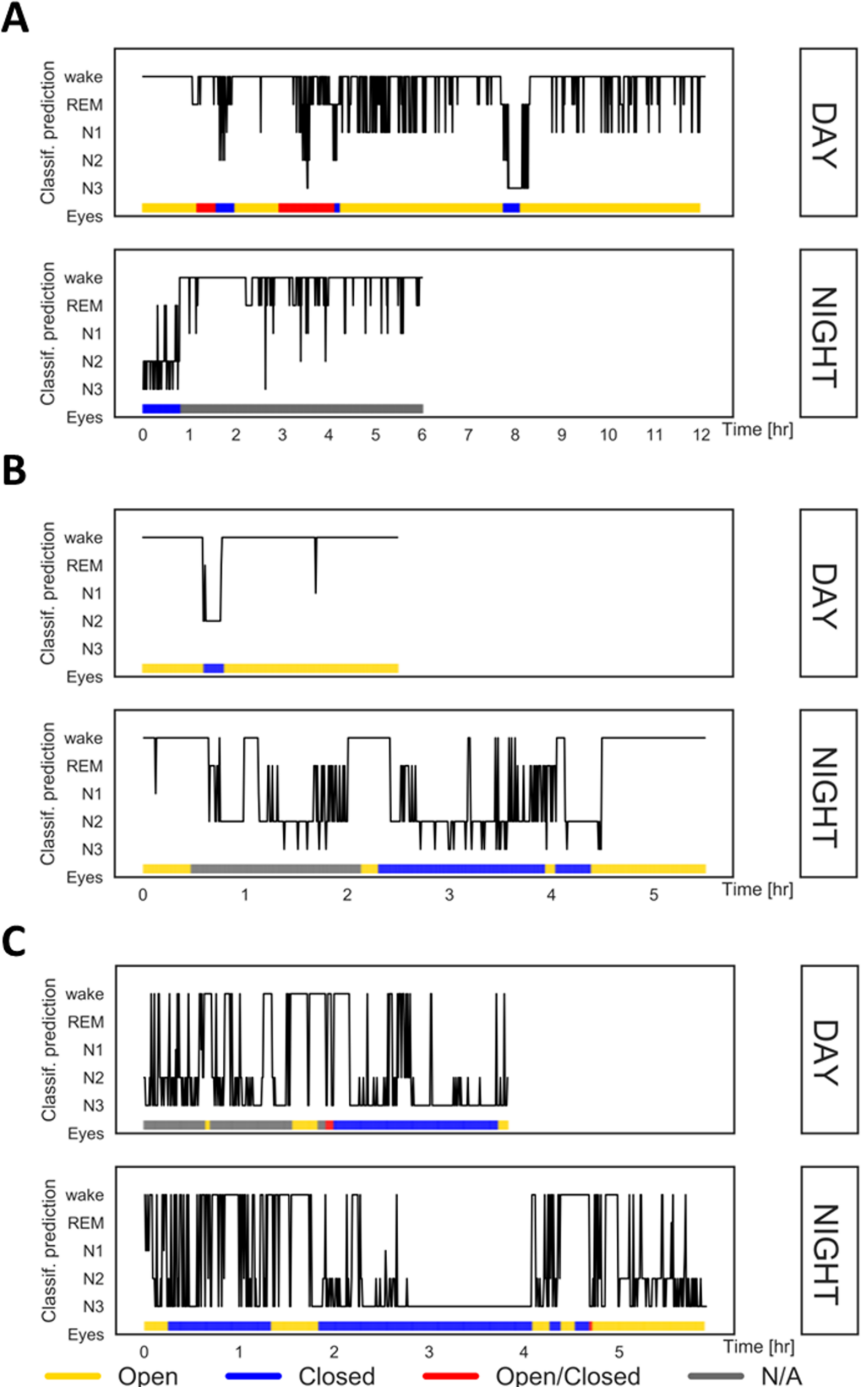
Classification results for day and night recordings in three exemplary patients. A = patient 1 (UWS), B = patient 3 (UWS), C = patient 7 (MCS). Classifier trained on healthy participants’ data is used for an epoch-by-epoch scoring (epochs plotted on the x-axis; 120 epochs = 1h). Predictions (plotted on the y-axis) are validated using simultaneous infrared video recordings. Open eyes: continuously open (≥5 minutes), Closed eyes: continuously closed (≥5 minutes), Open/Closed: switching between opening and closure, N/A–not applicable, i.e. not scorable.

After fitting the random forest classifier using data from healthy participants, the classifier was used for automatic epoch-by-epoch classification for each DOC patient. Results were evaluated by inspecting hypnograms as well as by computing a binary F1-score which quantifies the agreement between eyes being closed and the classifier predicting sleep (i.e., jointly N1, N2, N3, REM). The median F1-score for all subjects was 0.66 (MAD = 0.24). Out of the 23 subjects, only 4 subjects showed clear misclassification with a median F1-score of 0.1 (SD = 0.06). For those subjects the classifier predicted mainly stages N2 and N3 while eyes were opened or open/closed. Importantly, in 11 patients (5 MCS and 6 UWS) classification was highly accurate with a median F1-score of 0.87 (SD = 0.06), i.e. there was a strong overlap between the classifier predicting sleep (i.e. one of the four sleep stages) and the patients’ eyes being closed. Three exemplary cases are illustrated in Fig 4. These patients presented with extended periods of opened (e.g., patients 1 and 3) or closed eyes (e.g., patients 3 and 7) and demonstrate the overlap with our classification output as wake and N2/N3 sleep, respectively.

## Discussion

It is well-established, that a reliable characterization of sleep states in patients with disorders of consciousness might facilitate the overall assessment of a patient’s state and thus improve the diagnostic process [37]. Yet, direct application of standard sleep scorings schemes which rest on an ‘eyeball’ examination of EEG traces, such as scoring according to AASM criteria [14], provide highly inconsistent results (for review see; [38]), and requires highly experienced scorers [11]. Still, the inter-rater and probably even test-retest reliability will be low as one needs to flexibly adapt the criteria and form one’s own highly subjective rules (e.g., to reconcile contradicting evidence from the hemispheres or the undefined differentiation between an assumed pathological and a “real” slow wave) to manually classify sleep in the first place in these patients. We aimed to address these issues by developing an approach that can handle artefact-laden EEG signals and circumvents the insufficiency of standard heuristics for DOC sleep scoring by using machine learning based on permutation entropy [30]. Essentially, the prediction model was derived from a dataset from healthy individuals using the same scalp electrodes that had previously been sleep scored.

The results demonstrate that our classifier trained to identify 5 sleep stages on healthy data successfully generalized to a DOC dataset by yielding average (i.e. across subjects) accuracy of 0.63 (F-score), including 11 patients with high performance of 0.87 (F-score). Such cross-generalization, where classifier is trained on data from one group and successfully applied to other data indicates correspondence among neural patterns across these groups [39]. For only four patients it was impossible to classify sleep and wakefulness correctly (F-score < 0.13). Note, however, that the overall performance is likely to be underestimated as wake prediction for an awake patient having eyes closed will be considered as a misclassification. In other words, performance estimates are rather conservative as false negatives are not always false.

As a validation criterion we can only use the state of the eyes in our case as there is no verifiable “truth” for DOC wake/sleep staging. Measuring the classification performance using this behavioral criterion, the F1-score did not reveal any accuracy differences between UWS and MCS patients. One explanation might be that our validation criterion (i.e. eyes open/closed) is rather crude and only a binary approximation of the patients’ arousal, wherefore subtle differences in sleep architecture cannot be captured.

Thus, to overcome limitations of the behavioral validation we performed cluster analyses and compared the resulting patterns between groups (i.e. healthy, MCS, UWS). The results suggest that wakefulness appears more during the day whereas both REM-like and N2-like appear more at night in MCS patients (cf. Fig 2). In contrast, UWS patients do not show any accumulation of a specific state during either day or night. Although the classification pattern suggests that what we see in MCS patients relates to actual sleep stages, we cannot conclude to what extent classification in DOC reflects true sleep stages. However, the results suggest that certain periods are more N2-like, N3-like or REM-like.

Altogether the results suggest that we here present a highly data-driven approach to classify sleep/wake periods in DOC patients that may represent an alternative to the current rough and highly subjective estimation of sleep stages that relies on the state of the eyes, or on subjective criteria that are little consistent across publications. The small set of PSG channels needed (14) and the entropy transformation of our data make the recording of such data comparably easy and robust against artefacts.

In summary, the current approach is a first attempt to apply machine learning to such long-term data. We suggest that the approach will be used for further and higher performing classifiers. Finally, we speculate that the individual sleep stages derived for the patients may also predictive of the outcome of the patients, which remains to be tested.

## Supporting information

S1 Table Demographic data for patients. The analyzed patient sample 12 UWS and 11 MCS subjects. Abbreviations: M = male, F = female, TBI = Traumatic Brain Injury, CVA-Cerebrovascular Accident, SSPE = Subacute Sclerosing Panencephalitis, SD- = lower severe disability (3 points on Extended Glasgow Outcome Scale), eMCS = emergence from MCS; CRC-R = Coma Recovery Scale-Revised.
(PDF)

## References

[1] Pichler G, Fazekas F. Cardiopulmonary arrest is the most frequent cause of the unresponsive wakefulness syndrome: A prospective population-based cohort study in Austria. Resuscitation. 2016;103:94–8. doi: 10.1016/j.resuscitation.2016.02.023. WOS:000375898600029. 26980348 10.1016/j.resuscitation.2016.02.023

[2] Teasdale B, Jennett G. ASSESSMENT OF COMA AND IMPAIRED CONSCIOUSNESS: A Practical Scale. The Lancet. 1974;304(7872):81–4. 10.1016/s0140-6736(74)91639-0 4136544

[3] Giacino JT, Kalmar K, Whyte J. The JFK Coma Recovery Scale-Revised: Measurement characteristics and diagnostic utility. Archives of physical medicine and rehabilitation. 2004;85(12):2020–9. 15605342 10.1016/j.apmr.2004.02.033

[4] Laureys S, Celesia GG, Cohadon F, Lavrijsen J, León-Carrión J, Sannita WG, et al Unresponsive wakefulness syndrome: a new name for the vegetative state or apallic syndrome. BMC Medicine. 2010:8: 68 doi: 10.1186/1741-7015-8-68 21040571 10.1186/1741-7015-8-68 PMC2987895

[5] Giacino JT, Ashwal S, Childs N, Cranford R, Jennett B, Katz DI, et al The minimally conscious state: definition and diagnostic criteria. Neurology. 2002;58(3):349–53. . 11839831 10.1212/wnl.58.3.349

[6] Bergamasco B, Bergamini L, Doriguzzi T, Sacerdote I. The sleep cycle in coma: prognostic value. Electroencephalography and clinical neurophysiology. 1968;28(87). 4174810

[7] Chatrian GE, White LE, Daly D. Electroencephalographic patterns resembling those of sleep in certain comatose states after injuries to the head. Electroencephalography and clinical neurophysiology. 1963;15:272–80. 14020346 10.1016/0013-4694(63)90096-8

[8] Forgacs PB, Conte MM, Fridman EA, Voss HU, Victor JD, Schiff ND. Preservation of electroencephalographic organization in patients with impaired consciousness and imaging-based evidence of command-following. Ann Neurol. 2014;76(6):869–79. doi: 10.1002/ana.24283 ; PubMed Central PMCID: PMCPMC4354809. 25270034 10.1002/ana.24283 PMC4354809

[9] Blume C, Lechinger J, Santhi N, del Giudice R, Gnjezda MT, Pichler G, et al Significance of circadian rhythms in severely brain-injured patients: A clue to consciousness? Neurology. 2017;88(20):1933–41. doi: 10.1212/WNL.0000000000003942 ; PubMed Central PMCID: PMCPMC5444311. 28424270 10.1212/WNL.0000000000003942 PMC5444311

[10] Landsness E, Bruno MA, Noirhomme Q, Riedner B, Gosseries O, Schnakers C, et al Electrophysiological correlates of behavioural changes in vigilance in vegetative state and minimally conscious state. Brain. 2011;134(Pt 8):2222–32. doi: 10.1093/brain/awr152 ; PubMed Central PMCID: PMCPMC3155704. 21841201 10.1093/brain/awr152 PMC3155704

[11] Pavlov YG, Gais S, Muller F, Schonauer M, Schapers B, Born J, et al Night sleep in patients with vegetative state. J Sleep Res. 2017 doi: 10.1111/jsr.12524 . 28444788 10.1111/jsr.12524

[12] Wislowska M, Giudice RD, Lechinger J, Wielek T, Heib DP, Pitiot A, et al Night and day variations of sleep in patients with disorders of consciousness. Sci Rep. 2017;7(1):266 doi: 10.1038/s41598-017-00323-4 ; PubMed Central PMCID: PMCPMC5428269. 28325926 10.1038/s41598-017-00323-4 PMC5428269

[13] Cologan V, Drouot X, Parapatics S, Delorme A, Gruber G, Moonen G, et al Sleep in the unresponsive wakefulness syndrome and minimally conscious state. J Neurotrauma. 2013;30(5):339–46. doi: 10.1089/neu.2012.2654 . 23121471 10.1089/neu.2012.2654

[14] Iber C, Ancoli-Israel S, Chesson A, Quan S. American Academy of Sleep Medicine. The AASM Manual for the Scoring of Sleep and Associated Events: Rules, Terminology and Technical Specifications. Westchester: American Academy of Sleep Medicine; 2007.

[15] Rechtschaffen A, Kales A. A manual of standardized terminology, techniques and scoring system of sleep stages in human subjects. National Institutes of Health publication,; no 204 1968:57.

[16] Biase S, Gigli GL, Lorenzut S, Bianconi C, Sfreddo P, Rossato G, & al e. The importance of polysomnography in the evaluation of prolonged disorders of consciousness: sleep recordings more adequately correlate than stimulus-related evoked potentials with patients’ clinical status. Sleep Medicine. 2014;15:393–400. doi: 10.1016/j.sleep.2013.09.026 24508049 10.1016/j.sleep.2013.09.026

[17] Giubilei F, Formisano R, Fiorini M, Vitale A, Faroni J, Toni D, et al Sleep abnormalities in traumatic apallic syndrome. J Neurol Neurosurg Psychiatry. 1995;58(4):484–6. ; PubMed Central PMCID: PMCPMC1073441. 7738562 10.1136/jnnp.58.4.484 PMC1073441

[18] Nuwer M. Assessment of digital EEG, quantitative EEG, and EEG brain mapping: report of the American Academy of Neurology and the American Clinical Neurophysiology Society. Neurology. 1997;49(1):277–92. . 9222209 10.1212/wnl.49.1.277

[19] Malinowska U, Chatelle C, Bruno MA, Noirhomme Q, Laureys S, Durka PJ. Electroencephalographic profiles for differentiation of disorders of consciousness. Biomed Eng Online. 2013;12:109 doi: 10.1186/1475-925X-12-109 ; PubMed Central PMCID: PMCPMC3819687. 24143892 10.1186/1475-925X-12-109 PMC3819687

[20] Noirhomme Q, Lehembre R, Lugo Zdel R, Lesenfants D, Luxen A, Laureys S, et al Automated analysis of background EEG and reactivity during therapeutic hypothermia in comatose patients after cardiac arrest. Clin EEG Neurosci. 2014;45(1):6–13. doi: 10.1177/1550059413509616 . 24452769 10.1177/1550059413509616

[21] Anderer P, Gruber G, Paraptics S, Woertz M, Miazhynskaia T, Klösch G, et al An E-health solution for automatic sleep classification according to Rechtschaffen and Kales: validation study of the Somnolyzer 24 x 7 utilizing the Siesta database. Neuropsychobiology. 2005. 10.1159/000085205 15838184

[22] Jasper HH. The Ten-Twenty Electrode System of the International Federation. Electroencephalography and Clinical Neurophysiology. 1958;10:371–5. 10590970

[23] Gratton G, Coles MG, Donchin E. A new method for off-line removal of ocular artifact. Electroencephalogr Clin Neurophysiol. 1983;55(4):468–84. . 6187540 10.1016/0013-4694(83)90135-9

[24] Akerstedt T, Gillberg M. Subjective and objective sleepiness in the active individual. Int J Neurosci. 1990;52(1–2):29–37. . 2265922 10.3109/00207459008994241

[25] Staniek M, Lehnertz K. Symbolic transfer entropy. Phys Rev Lett. 2008;100(15):158101 doi: 10.1103/PhysRevLett.100.158101 . 18518155 10.1103/PhysRevLett.100.158101

[26] Olofsen E, Sleigh JW, Dahan A. Permutation entropy of the electroencephalogram: a measure of anaesthetic drug effect. Br J Anaesth. 2008;101(6):810–21. doi: 10.1093/bja/aen290 . 18852113 10.1093/bja/aen290

[27] Jordan D, Stockmanns G, Kochs EF, Pilge S, Schneider G. Electroencephalographic order pattern analysis for the separation of consciousness and unconsciousness: an analysis of approximate entropy, permutation entropy, recurrence rate, and phase coupling of order recurrence plots. Anesthesiology. 2008;109(6):1014–22. doi: 10.1097/ALN.0b013e31818d6c55 . 19034098 10.1097/ALN.0b013e31818d6c55

[28] King JR, Sitt JD, Faugeras F, Rohaut B, El Karoui I, Cohen L, et al Information sharing in the brain indexes consciousness in noncommunicative patients. Curr Biol. 2013;23(19):1914–9. doi: 10.1016/j.cub.2013.07.075 . 24076243 10.1016/j.cub.2013.07.075 PMC5635964

[29] Thul A, Lechinger J, Donis J, Michitsch G, Pichler G, Kochs EF, et al EEG entropy measures indicate decrease of cortical information processing in Disorders of Consciousness. Clin Neurophysiol. 2016;127(2):1419–27. doi: 10.1016/j.clinph.2015.07.039 . 26480834 10.1016/j.clinph.2015.07.039

[30] Bandt C, Pompe B. Permutation entropy: a natural complexity measure for time series. Phys Rev Lett. 2002;88(17):174102 doi: 10.1103/PhysRevLett.88.174102 . 12005759 10.1103/PhysRevLett.88.174102

[31] Dalton L, Ballarin V, Brun M. Clustering algorithms: on learning, validation, performance, and applications to genomics. Curr Genomics. 2009;10(6):430–45. doi: 10.2174/138920209789177601 ; PubMed Central PMCID: PMCPMC2766793. 20190957 10.2174/138920209789177601 PMC2766793

[32] Hastie T, Tibshirani R, Friedman J. The Elements of Statistical Learning Data Mining, Inference, and Prediction. Second Edition ed. Springer, editor. New York 2016.

[33] Sokolova M, Lapalme G. A systematic analysis of performance measures for classification tasks. Information Processing and Management. 2009;45. Epub 437.

[34] Team RC. R: A language and environment for statistical computing. R Foundation for Statistical Computing Vienna, Austria 2013.

[35] Galili T. dendextend: an R package for visualizing, adjusting, and comparing trees of hierarchical clustering. 2015 doi: 10.1093/bioinformatics/btv428 26209431 10.1093/bioinformatics/btv428 PMC4817050

[36] Kuhn M. caret: Classification and Regression Training. 6.0–76 ed2017.

[37] Cologan V, Schabus M, Ledoux D, Moonen G, Maquet P, Laureys S. Sleep in disorders of consciousness. Sleep Med Rev. 2010;14(2):97–105. doi: 10.1016/j.smrv.2009.04.003 ; PubMed Central PMCID: PMCPMC2855378. 19524464 10.1016/j.smrv.2009.04.003 PMC2855378

[38] Blume C, Del Giudice R, Wislowska M, Lechinger J, Schabus M. Across the consciousness continuum-from unresponsive wakefulness to sleep. Front Hum Neurosci. 2015;9:105 doi: 10.3389/fnhum.2015.00105 ; PubMed Central PMCID: PMCPMC4354375. 25805982 10.3389/fnhum.2015.00105 PMC4354375

[39] Grootswagers T, Wardle SG, Carlson TA. Decoding Dynamic Brain Patterns from Evoked Responses: A Tutorial on Multivariate Pattern Analysis Applied to Time Series Neuroimaging Data. J Cogn Neurosci. 2017;29(4):677–97. doi: 10.1162/jocn_a_01068 . 27779910 10.1162/jocn_a_01068

