# Supplementary material for: Sleep in patients with disorders of consciousness characterized by means of machine learning
Source: PLoS One. 2018 Jan 2;13(1):e0190458. doi: 10.1371/journal.pone.0190458 (PMC5749793; doi:10.1371/journal.pone.0190458)
Supplement: S1 Table — The analyzed patient sample 12 UWS and 11 MCS subjects. Abbreviations: M = male, F = female, TBI = Traumatic Brain Injury, CVA-Cerebrovascular Accident, SSPE = Subacute Sclerosing Panencephalitis, SD- = lower severe disability (3 points on Extended Glasgow Outcome Scale), eMCS = emergence from MCS; CRC-R = Coma Recovery Scale-Revised. (PDF) [file pone.0190458.s001.pdf]

Nb	Age (years)	Sex	Aetiology	Period since injury (months)	Clinical condition	Clinical assessment	CRS-R total score	Follow-up diagnosis	Period between recording and follow-up (months)
1	74	F	TBI	1	sub-acute	UWS	3	death	24
2	19	M	SSPE	24	chronic	UWS	3	-	-
3	52	M	TBI	13	chronic	UWS	4	MCS	14
4	58	F	CVA	28	chronic	UWS	4	death	-
5	50	F	CVA	45	chronic	UWS	4	-	-
6	62	M	CVA	1	chronic	UWS	4	death	1
7	61	M	anoxia	32	chronic	UWS	4	death	-
8	54	M	anoxia	9	chronic	UWS	5	death	10
9	21	M	TBI	7	sub-acute	UWS	6	UWS	150
10	16	M	TBI	21	chronic	UWS	6	-	-
11	61	F	CVA	1	sub-acute	UWS	6	SD-	12
12	16	F	TBI	1	sub-acute	UWS	7	SD-	12
13	45	M	TBI	12	sub-acute	MCS	8	eMCS	24
14	62	M	TBI	2	sub-acute	MCS	8	-	-
15	34	M	anoxia	240	chronic	MCS	8	-	-
16	66	M	CVA	3	sub-acute	MCS	10	-	-
17	61	M	anoxia	2	sub-acute	MCS	10	-	-
18	48	M	TBI	8	sub-acute	MCS	11	death	-
19	31	F	CVA	1.5	sub-acute	MCS	11	-	-
20	21	M	anoxia	28	chronic	MCS	13	MCS	20
21	30	M	TBI	120	chronic	MCS	13	MCS	67
22	50	F	TBI	113	chronic	MCS	14	-	-
23	43	F	TBI	6	sub-acute	MCS	21	-	-
